# Sensitivities of Key Parameters in the Preparation of Silver/Silver Chloride Electrodes Used in Harned Cell Measurements of pH

**DOI:** 10.3390/s110808072

**Published:** 2011-08-17

**Authors:** Paul J. Brewer, Daniela Stoica, Richard J. C. Brown

**Affiliations:** 1 Analytical Science Division, National Physical Laboratory, Teddington, Middlesex TW11 0LW, UK; E-Mail: richard.brown@npl.co.uk; 2 Department of Biomedical and Inorganic Chemistry, Laboratoire National de Métrologie et d’Essais, 1 rue G. Boissier, 75015 Paris, France; E-Mail: daniela.stoica@lne.fr

**Keywords:** pH, Harned cell, Ag/AgCl electrode, primary methods

## Abstract

A questionnaire was completed by fourteen world leading national metrology institutes to study the influence of several variables in the preparation of Ag/AgCl electrodes on the accuracy of Harned cell measurements of pH. The performance of each institute in the last decade has been assessed based on their results in eight key comparisons, organized by the Bureau International des Poids et Measures Consultative Committee for Amount of Substance, involving the measurement of pH of phosphate, phthalate, carbonate, borate and tetroxalate buffer solutions. The performance of each laboratory has been correlated to the results of the questionnaire to determine the critical parameters in the preparation of Ag/AgCl electrodes and their sensitivities with respect to the accuracy of pH measurement. This study reveals that the parameters most closely correlated to performance in comparisons are area of electrode wire exposed to the electrolyte, diameter and porosity of the Ag sphere prior to anodisation, amount of Ag converted to AgCl during anodisation, stability times employed for electrodes to reach equilibrium in solution prior to measurement, electrode rejection criteria employed and purity of reagents.

## 1. Introduction

The concept of pH was introduced by Sørensen in 1909 [1]. Since then, measurements of pH have become increasingly widespread. Today, pH is amongst the most frequently measured quantities and impacts on a whole slew of different market sectors [2–5]. Due to the significant economic and societal consequences of measurement inaccuracies it is imperative to ensure validity and traceability [6–8].

Primary pH values are determined using an electrochemical cell arrangement which is referred to as the “Harned cell”. The cell which does not contain a liquid junction relies on well characterised Ag/AgCl reference electrodes for operation [9]. It has the potential to be a primary method for the absolute measurement of pH, providing that it can conform to the accepted definition of a primary method [10] that requires a methodology and operation that can be completely described and understood, for which a complete uncertainty statement can be written down in terms of SI units.

A large number of National Metrology Institutes (NMIs) have developed a capability to underpin pH measurement using the Harned cell. The estimated uncertainty of pH determined using the primary methods developed at these institutes is approximately 0.002 (*k* = 2) [9]. However in international comparisons, the comparability between institutes is usually poorer than 0.005 (*k* = 2).

The performance of Ag/AgCl reference electrodes is of paramount importance for accurate pH measurement. A small change in the potential of a Ag/AgCl reference electrode makes a significant contribution to the measurement uncertainty. Usually, in metrological applications, excluding the determination of the molality of HCl used in the Harned cell, the reference potential of Ag/AgCl electrodes employed is the largest contribution to the measurement uncertainty of pH [9]. Ag/AgCl electrodes are usually prepared at NMIs, following procedures developed by standardisation bodies or bespoke methods developed in house [11–14]. NMIs have gained a wealth of experience in electrode preparation from investing a significant effort into refining methods to improve performance [15]. However the sensitivities of various processes in the procedures and their impact on electrode performance are still poorly understood and may account for differences between reference values submitted by NMIs in key comparisons. Differences still exist in methods of preparation and in the way similar methods are implemented. To date, a detailed investigation has not been carried out.

The aim of this study is to improve performance of Ag/AgCl electrodes by understanding the sensitivities of various parameters involved in the manufacturing process. This study for the first time identifies differences in preparation techniques between NMIs in order to understand the causes of any discrepancies in the standard potential of electrodes produced. It is an important step towards understanding the sensitivities in the preparation method on repeatability of the electrode potential and performance. The intention of this work is to improve future international comparability in pH measurement.

The results from eight key comparisons (K9 and K9.2, K17, K18 and K18.1, K19 and K19.1 and K20 involving measurement of phosphate, phthalate, carbonate, borate and tetroxalate buffer materials respectively), organized by the Bureau International des Poids et Measures (BIPM) Consultative Committee for Amount of Substance (CCQM), have been used as an indicator of NMI performance.

## 2. Experimental Section

The Ag/AgCl electrodes studied in this work were prepared at the fourteen NMIs using variations of published literature procedures for the fabrication of the thermal electrolytic type reference electrodes [11–14]. This procedure was achieved by thermal decomposition of several separate applications of Ag_2_O paste to a Pt or Ag wire. Following this process, 5–25% of the material was electrolytically converted to AgCl in a solution of either 0.1 or 1 M HCl.

A questionnaire of 57 questions was constructed and sent to all NMIs with capability in pH measurement. As a result of the replies received a supplementary questionnaire was prepared to capture additional parameters and sensitivities. The questions resulting in the most variable responses were examined further in Section 3.

## 3. Results and Discussion

### 3.1. Correlating Degree of Equivalence in Key Comparisons of pH to Ag/AgCl Electrode Performance

**Figure 1.**
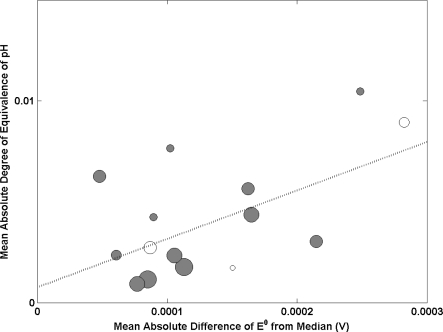
Correlating degree of equivalence in key comparisons of pH to Ag/AgCl electrode performance. A line of best fit has been added to the graph which has been weighted by an NMI’s participation. The white circles indicate the NMI’s which have not participated in this study.

Eight international key comparisons involving pH measurements have been run in the last decade. In each comparison, the results from each laboratory are characterized in terms of a ‘degree of equivalence’ (DOE) representing the deviation of its result from the accepted Key Comparison Reference Value (KCRV). Figure 1 shows the results of plotting the mean DOE against the mean absolute difference of E^0^ from the median (used as an indicator of electrode performance) for the eight key comparisons of pH. Each sphere represents a different NMI with size being proportional to participation in key comparisons (the larger the sphere, the more key comparisons an NMI has participated in). A large mean absolute DOE of pH was observed for one NMI and was deemed as an outlier. Data from this institute has not been included in the analysis.

The data shows a positive correlation between these two variables and suggests that institutes with the capability to produce high performance Ag/AgCl electrodes (small deviation of E^0^ from the median) have good results in key comparisons of pH (demonstrated by a small mean DOE). There are some outliers to the line of best fit (weighted by participation). However with the exception of one, these NMIs have participated in 3 or fewer key comparisons. Hence one could argue that there is insufficient data to observe a correlation for these institutes. One of the outliers has participated in 5 key comparisons and demonstrates a capability to produce high performing Ag/AgCl electrodes with a poor mean performance in key comparisons. In this case there is likely to be other factors influencing their pH measurement capability. This analysis demonstrates the impact of Ag/AgCl electrode performance on comparability in pH measurement.

There is some argument that the slope of the acidity function (a measure of the acidity of a medium or solvent) might be a better indicator of electrode performance than E^0^ as standard potentials will cancel out in pH measurement (assuming electrode stability) while the slope is assumed to have a theoretical value. However a drawback is that the uncertainty in determining the slope is often large due to large residuals of the data from a straight line fit. In addition, data on the slope of the acidity function from key comparisons is often not published and therefore not as readily available as E^0^ values. In this study we use absolute E^0^ values and make the assumption that electrodes exhibiting non ideal E^0^ (due to impurities *etc*.) are also likely to exhibit similarly non-ideal slopes.

It is clear from the outliers in Figure 1, that there are some parameters other than the performance of Ag/AgCl electrodes that affect the DOE in the key comparisons. By comparing parameters in the preparation of Ag/AgCl electrodes to E^0^, we are able to de-convolute all other factors contributing uncertainty to the measurement of pH. Also, given that a good correlation exists between E^0^ and DOE in most cases, E^0^ can be taken to act as a surrogate for an NMI’s performance in pH comparisons. Due to the often randomly scattered nature of the data reported in comparisons, absolute values of DOE and E^0^ were used so that the calculated mean of all comparisons represented the average deviation of an NMI from the reference value and the median of E^0^. The advantage of this is that random variations are captured, rather than summing to zero.

The results which follow feature responses to questions that received fewer than 5 identical responses from the fourteen participating laboratories and show a good correlation to the mean absolute difference of E^0^ from the median of the eight key comparisons. The remainder of questions from this study received larger agreement between laboratories and for this reason the sensitivity of these parameters could not be investigated further.

### 3.2. Physical Characteristics

**Figure 2.**
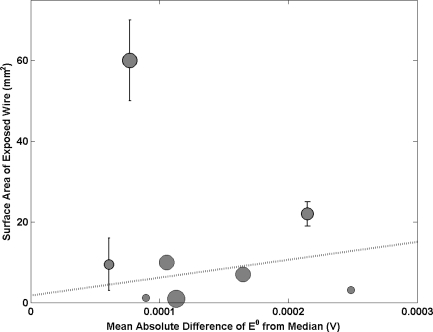
Surface area of the electrode wire that is exposed to the electrolyte as a function of the mean absolute difference E^0^ from the median. Each sphere represents a different NMI with size being proportional to amount of participation in key comparisons. Bars on the data points indicate that a range of values were submitted. A line of best fit has been added and is weighted by an NMI’s participation. The anomalous data point at 60 mm^2^ has been removed from the fit.

Ag/AgCl electrodes are prepared by coating a wire (often Pt) which protrudes from the glass electrode body with Ag_2_O. This material is then converted to Ag/AgCl following a 2 stage process. The fabrication process often leaves some wire between the Ag/AgCl material and glass body exposed. Figure 2 shows a positive correlation between the surface area of electrode wire that is exposed to the electrolyte with electrode performance (mean deviation of E^0^ from the median). The graph suggests that as the surface area of exposed wire is reduced, the electrode performance improves. One NMI is anomalous to this observation with a large surface area of wire exposed (50–70 mm^2^) and good performance (mean absolute difference of E^0^ from median < 0.0001 V). This NMI produces electrodes from Ag wire where all other institutes use Pt. With Pt exposed to the electrolyte, one would expect a mixed potential to be set up, hence causing a deviation in the value of E^0^ (the correlation observed supports this). Electrodes made from Ag wire would not suffer from this effect as Ag makes up the majority of the coated sphere. This accounts for why good electrode performance was achieved with a large amount of wire exposed to the electrolyte. Hence, when using Pt wire it is important to keep the exposed area of wire to a minimum.

**Figure 3.**
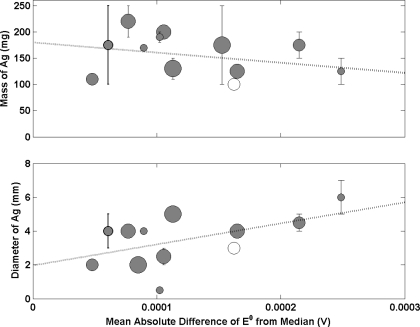
**(a)** Mass and **(b)** diameter of Ag sphere prior to conversion to Ag/AgCl, each as a function of the mean absolute difference of E^0^ from the median. Each sphere represents a different NMI with size being proportional to amount of participation in key comparisons. Bars on the data points indicate that a range of values were submitted. A line of best fit has been added to the data (weighted by participation). White circles indicate laboratories that have submitted less than values (and have not been included in the line of best fit).

Figure 3(a) shows the relationship between the mass of Ag added to produce an electrode (prior to conversion to Ag/AgCl) and the mean absolute difference of E^0^ from the median. The data shows no obvious correlation. The weighted line of best fit tentatively suggests a tendency for smaller Ag spheres to result in poorer electrodes. However, the slope is very small showing no obvious relationship between electrode performance and mass of Ag. Interestingly, when mass is substituted for diameter of the Ag sphere deposited (Figure 3(b)), a positive correlation is exhibited with larger spheres resulting in poorer electrode performance. This effect has been described in recent work [16,17] and is due to the presence of a microporous structure that limits the rate at which traces of any previous solutions are diluted by any new environment. Larger diameter spheres of Ag/AgCl were shown to require longer times to reach equilibrium which is consistent with the process being described by diffusion whereby traces of the previous solution diffuses out of the pore structure while the new solution diffuses in. This increases the probability of contaminating the solution with a previous storage/measurement solution. The observed correlation in Figure 3(b) supports this theory and highlights the requirement to produce electrodes with spheres of smaller diameter. A correlation between mass and diameter of the Ag sphere would be expected. However, the fact that Figure 3(a) does not show the same trend as Figure 3(b) suggests that the porosity of the Ag/AgCl material may differ. Data from the 2 NMIs with the poorest electrodes produce Ag spheres with large diameters compared to the others but with a similar mass of material. This would suggest the Ag/AgCl material contains a larger degree of porosity. The performance may be compromised due to increased equilibrium times and solution contamination as a result of a more porous structure.

**Figure 4.**
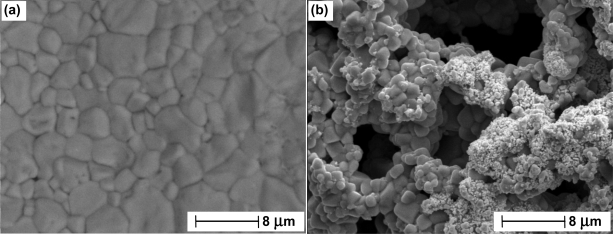
SEM images of the spherical Ag/AgCl material in thermal electrolytic Ag/AgCl electrodes prepared at two different NMIs **(a)** and **(b)**.

A large amount of work using electron microscopy has been carried out to study the porosity of thermal electrolytic Ag/AgCl electrodes [18,19]. The work suggests a structure with a high degree of porosity. Recent work has been conducted to study the structure of two thermal electrolytic Ag/AgCl electrodes prepared at 2 different NMIs. The SEM images in Figure 4 show a stark difference in porosity between the two structures. The electrode produced by the NMI in Figure 4(a) reveals little pore structure. In the context of Figure 3, this would represent one of the results showing a lower than average diameter with higher than average mass. The electrode shown in Figure 4(b) shows a large degree of porosity and would represent a lower than average mass with larger than average diameter in Figure 3. As mentioned in previous work [19,20], for optimum electrode performance, the geometric surface area (related to degree of porosity and diameter) should be large enough to ensure a high absolute exchange current at equilibrium to enable good long term stability whilst having a small enough diameter so as to reduce the time it takes an electrode to reach equilibrium.

**Figure 5.**
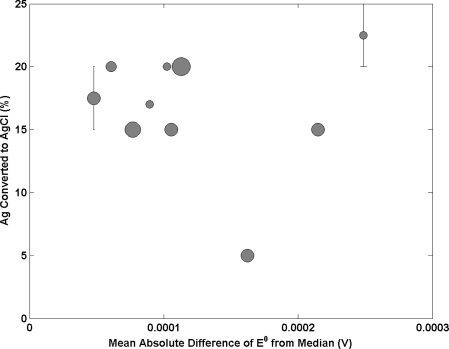
Amount of Ag converted to AgCl as a function of the mean absolute difference E^0^ from the median. Each sphere represents a different NMI with size being proportional to amount of participation in key comparisons. Bars on the data points indicate that a range of values were submitted.

Figure 5 reports the influence of altering the content of Ag that is converted to AgCl on electrode performance. The data suggests an optimum at around 15–20% AgCl exists with laboratories converting higher and lower amounts of Ag to AgCl than this range producing poorer electrodes. The best performing lab uses 17%, a cluster at 20% and 15% show intermediate performance and the poorest performance electrodes contain 5% AgCl in one case, 20–25% in another and one at 15%. However the data provided is not enough to strongly support this statement as the uncertainty in the AgCl content reported is expected to be large. Further research is required to understand the relationship between electrode composition and electrode performance.

**Figure 6.**
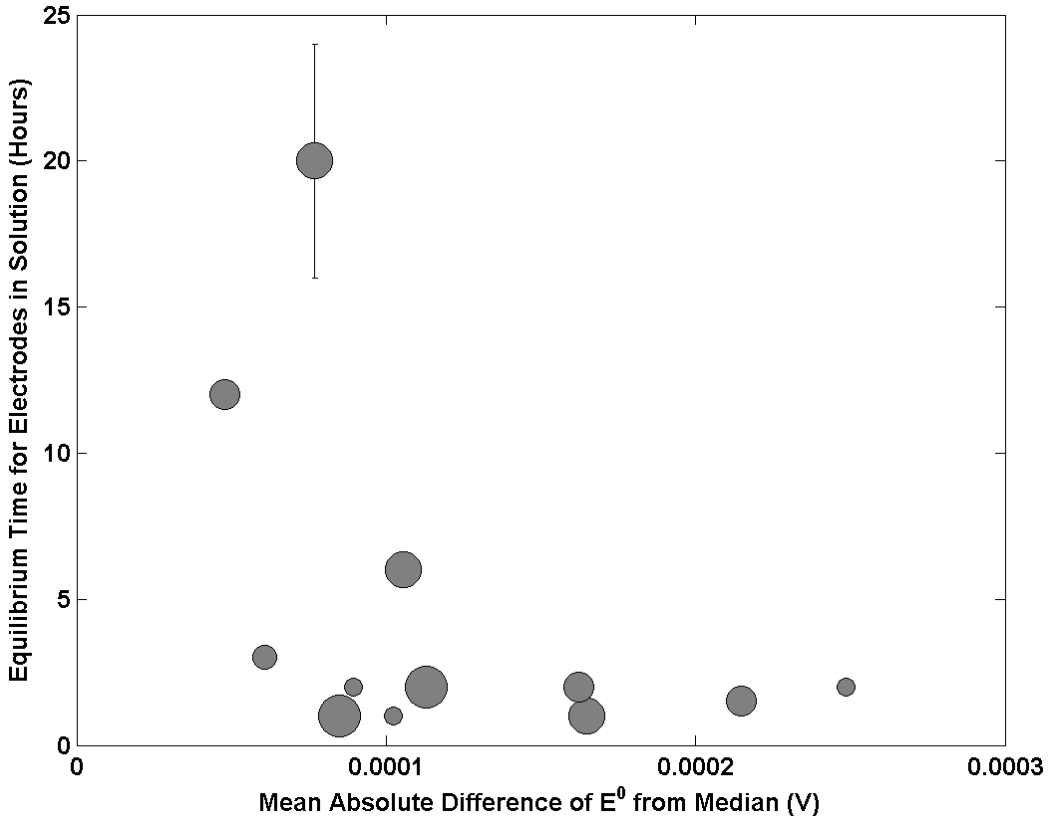
Time electrodes are in solution prior to measurement as a function of the mean absolute difference of E^0^ from the median. Each sphere represents a different NMI with size being proportional to amount of participation in key comparisons. Bars indicate a range of times were employed.

Figure 6 presents the results of the time in which an electrode is allowed to reach equilibrium in solution prior to measurement. The data exhibits an interesting trend with the smallest E^0^ deviation from the median occurring for NMIs that employ longer equilibrium times. This relationship sharply plateaus for wait times below 3 hours (which the majority of institutes use). The data suggests a requirement to adopt longer equilibration times prior to measurement. This will be dependent on the porosity and diameter of the Ag/AgCl material with longer times being required for higher degrees of porosity and larger diameters. The solution the electrode was stored in prior to transfer to the Harned cell will also have an effect. This was addressed in this study. The majority of NMI’s use a low concentration of HCl solution (*i.e.*, 0.01 mol kg^−1^). However some use saturated AgCl solution.

### 3.3. Electrode History

**Figure 7.**
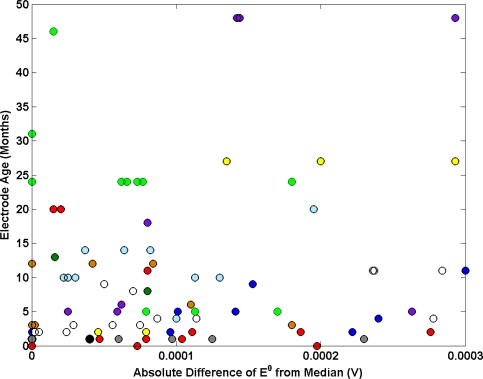
Electrode age as a function of the mean absolute difference of E^0^ from the median. The results from each comparison are plotted and each NMI is represented with a different colour.

An investigation into the impact of electrode history on electrode performance is presented in Figure 7. The age of a batch of Ag/AgCl electrode used in each key comparison is plotted as a function of the mean absolute difference of E^0^ from the median. Generally the data suggests no correlation between electrode age and performance.

### 3.4. Electrode Rejection Criterion

**Figure 8.**
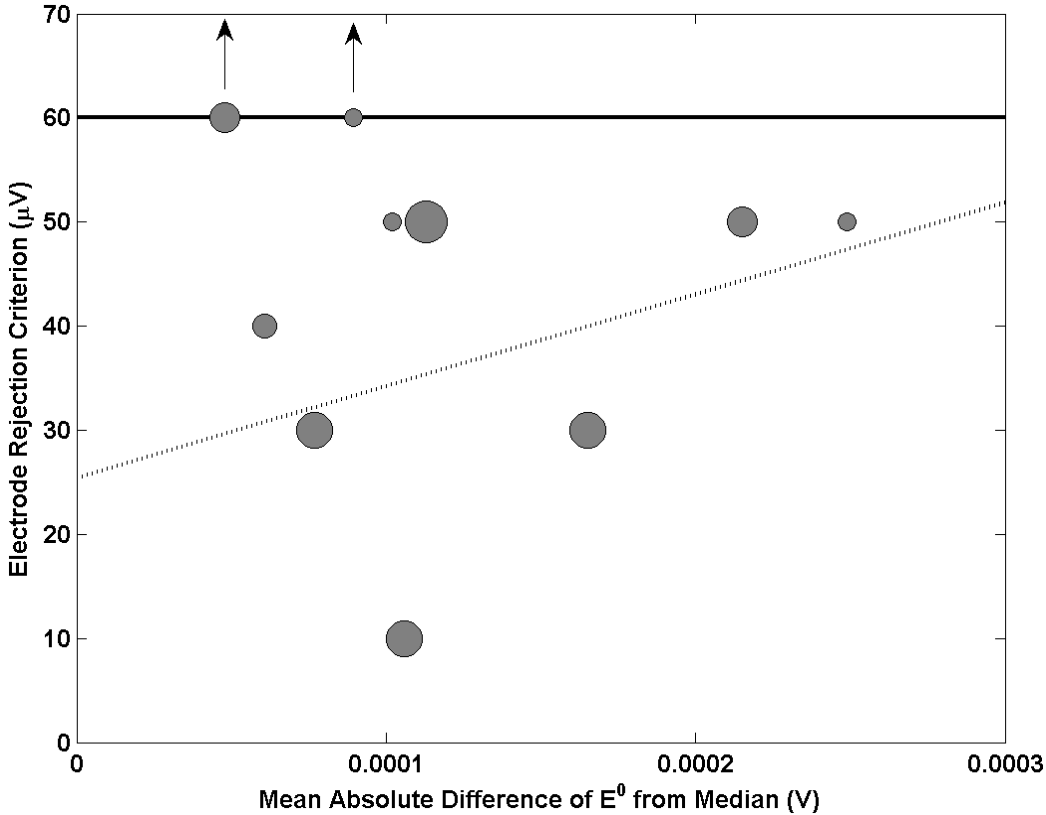
Electrode rejection criterion as a function of the mean absolute difference of E^0^ from the median. Each sphere represents a different NMI with size being proportional to amount of participation in key comparisons. The graph has been scaled to exclude the results of 2 NMI’s that were substantially larger than the others (these are indicated on the solid line with arrows and represent rejection criteria of 250 and 5,000 μV from left to right). A line of best fit has been added to the graph which has been weighted by an NMI’s participation.

The long and short term stability of Ag/AgCl reference electrodes is of paramount importance for accurate pH measurement as a small change in the potential of a Ag/AgCl reference electrode makes a significant contribution to the measurement uncertainty. Therefore in an attempt to select a set of similar reference electrodes and reduce the risk of changes in potential between electrodes, it is common practice to reject individual reference electrodes which differ from the average of the group by more than 100 micro volts [21]. Figure 8 shows the relationship between this rejection criterion employed at each NMI and electrode performance. A linear relationship is observed with electrode performance improving when the criterion is more stringent. This outlines the importance of selecting a set of electrodes with a small in which the standard deviation of their potential difference is small.

### 3.5. Further Observations

From the data provided there is some evidence that the purity of reagents used in the preparation of Ag_2_O (such as AgNO_3_ and NaOH) and the purity of the electrode wire influenced electrode performance. However, most data provided was taken from assays quoted on purchased reagents (often indicated with greater than values). Therefore for a thorough analysis to be performed, laboratories would be required to measure the impurities which is outside the scope of this study.

Two laboratories have reported a different procedure to the conventional anodisation, for converting some of the Ag to AgCl. The convertion is achieved by reacting Ag with Cl_2_ using a 0.01M HCl solution saturated with Cl_2_. The reason provided by the laboratory for adopting this alternative method is that it produces a more homogenous layer. However, there is not enough data to determine its effect on electrode performance. Similarly, one institute reports a different route to preparing Ag (Ag_2_CO_3_ paste is prepared from AgNO_3_ and NaHCO_3_ instead of Ag_2_O from AgNO_3_ and NaOH). The reason for this is that it is believed that HCO_3_^−^ does not adhere to the precipitate as much as OH^−^. A further alteration to the conventional method for producing Ag_2_O is that one institute uses KOH instead of NaOH.

Three institutes state that they apply thinner final Ag_2_O layers when coating the electrode. This is believed to result in a more uniform morphology of the final Ag/AgCl sphere. However, once again further work is required to establish its influence on electrode performance.

## 4. Conclusions

A questionnaire was completed by 14 participants to determine the critical parameters in the preparation of Ag/AgCl reference electrodes and their sensitivities with respect to the accuracy of pH measurement. This study reveals that the parameters most closely correlated to performance in comparisons are area of electrode wire exposed to the electrolyte, diameter and porosity of the Ag sphere prior to anodisation, amount of Ag converted to AgCl during anodisation, equilibrium time for electrodes in solution prior to measurement and electrode rejection criteria employed. From the data provided there is some evidence that the purity of reagents used in the preparation of Ag_2_O (such as AgNO_3_ and NaOH) and the purity of the electrode wire influenced electrode performance. Although the influence of several of these parameters on Ag/AgCl electrode performance might be expected [16,17,21–24], this study provides for the first time, information on the sensitivities of these parameters on repeatability of the electrode potential and performance at the international level, and how variations between institutes may affect the accuracy of primary pH measurement. Although this study is qualitative, it highlights a clear correlation for a number of parameters to performance in key comparisons. This allows for a unified strategy within the CCQM Working Group on Electrochemical Analysis for electrode design (even if a systematic bias still exists in E^0^ it will be constant across all NMIs) and may lead to improved future international comparability in pH measurement. This study may even have implications for the use of Ag/AgCl electrodes in emerging new areas of electrochemical research [25].
